# pH-dependent rGO/Cu–Cu_2_O electrodes with a porous PVA/PEO film for high-sensitivity non-enzymatic glucose sensing: a COMSOL multiphysics study

**DOI:** 10.1039/d5ra09249c

**Published:** 2026-03-31

**Authors:** Farag M. A. Altalbawy, Qusay Abdulsattar Mohammed, Huda Kadhim Jaafar, Normurot Fayzullaev, Manoj A. Vora, Subbulakshmi Ganesan, Renu Sharma, Geeta Durga, Pallavi Sharma, Shayan Amiri

**Affiliations:** a Department of Chemistry, University College of Duba, University of Tabuk Tabuk Saudi Arabia; b College of Dentistry, University of Al Maarif Al Anbar 31001 Iraq; c College of Applied Sciences, University of Technology-Baghdad Baghdad Iraq; d Department of Polymer Chemistry and Chemical Technology, Samarkand State University Samarkand 140101 Uzbekistan; e Department of Chemistry, Faculty of Science, Gokul Global University Sidhpur Gujarat India; f Department of Chemistry and Biochemistry, School of Sciences, JAIN (Deemed to be University) Bangalore Karnataka India; g Department of Chemistry, University Institute of Sciences, Chandigarh University Mohali Punjab India; h Department of Chemistry and Biochemistry, Sharda School of Engineering & Sciences, Sharda University Greater Noida India; i Centre for Research Impact & Outcome, Chitkara University Institute of Engineering and Technology, Chitkara University Rajpura Punjab 140401 India; j Department of Chemistry, Technical Engineering College, The Islamic University Najaf Iraq; k Lloyd Institute of Engineering & Technology Knowledge Park II Greater Noida Uttar Pradesh 201306 India; l Young Researchers and Elite Club, Tehran Branch, Islamic Azad University Tehran Iran sh.amiriacademic@gmail.com

## Abstract

A comprehensive 2D finite-element model based on COMSOL Multiphysics has been developed to investigate the pH-dependent electrochemical performance of reduced graphene oxide/copper–cuprous oxide (rGO/Cu–Cu_2_O) nanocomposite electrodes stabilised by a NaOH-treated porous polyvinyl alcohol/polyethylene oxide (PVA/PEO) film for non-enzymatic glucose detection in alkaline media (pH 9.12–14.09). The model couples the Nernst equation for open-circuit potential, Butler–Volmer kinetics for glucose oxidation *via* the Cu(ii)/Cu(iii) redox shuttle, Nernst–Planck transport for glucose and OH^−^, and charge conservation across the porous polymer layer. Optimal electrocatalytic activity is achieved at pH 13.03, delivering an ultrahigh sensitivity of 853.19 µA mM^−1^ cm^−2^, a stable open-circuit potential of 0.653 V (*vs.* Ag/AgCl), a linear range up to 10.2 mM, and a rapid response time of 2.08 s. Systematic parametric analysis reveals that decreasing PVA/PEO film thickness to ∼300 nm, reducing Cu–Cu_2_O nanoparticle diameter below 30 nm, and increasing rGO conductivity above 1400 S m^−1^ dramatically enhance both sensitivity and response speed by improving ion accessibility and electron-transfer efficiency. Model predictions are rigorously validated against experimental electrochemical impedance spectroscopy data (RMSE = 0.08), confirming predictive accuracy. The work elucidates fundamental pH–structure–performance relationships and provides quantitative design guidelines for robust, cost-effective, enzyme-free glucose sensors suitable for diabetes monitoring and wearable diagnostic platforms.

## Abstract 2

rGO/Cu–Cu₂O electrode optimized at pH 13.03 for enzyme-free glucose sensing.

## Graphical abstract

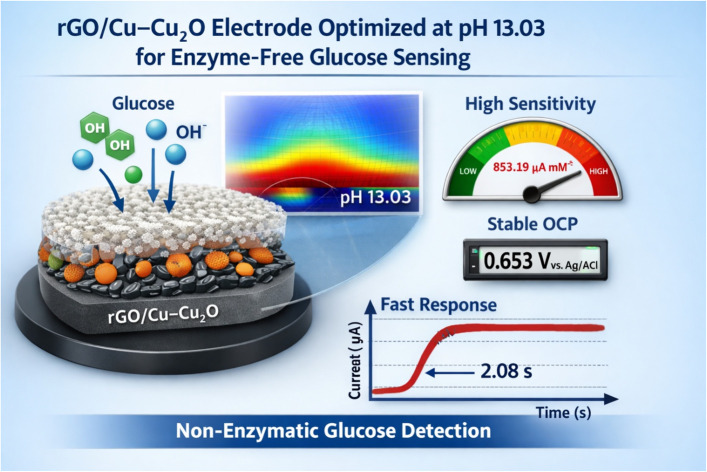

## 1. Introduction

Non-enzymatic glucose sensors have emerged as a transformative technology in electrochemical biosensing, addressing critical limitations of enzymatic sensors, such as enzyme degradation, sensitivity to environmental factors like temperature and pH, and high production costs.^1,2^ These sensors exploit the electrocatalytic properties of advanced materials, particularly transition metal-based nanocomposites, to achieve superior sensitivity, selectivity, and durability for glucose detection in biomedical and diagnostic applications.^3,4^ Among these materials, reduced graphene oxide (rGO) combined with copper–copper oxide (Cu–Cu_2_O) nanocomposites has attracted significant attention due to the synergistic interplay of rGO's high electrical conductivity (∼1000 S m^−1^) and Cu–Cu_2_O's robust catalytic activity for glucose oxidation.^5,6^ The incorporation of NaOH-treated polyvinyl alcohol/polyethylene oxide (PVA/PEO) polymeric films enhances electrode performance by preventing nanoparticle aggregation, thereby maintaining structural integrity and catalytic site accessibility in harsh alkaline environments.^7,8^ This study employs computational modeling to investigate the pH-dependent electrochemical performance of rGO/Cu–Cu_2_O nanocomposite electrodes, focusing on the complex interactions between material properties, such as rGO conductivity and nanoparticle size, and alkaline conditions (pH 9.12–14.09). By simulating electrochemical behavior, this work aims to elucidate optimal design parameters for non-enzymatic glucose sensors, offering insights into improving their practical applicability.

Accurate glucose detection is essential for managing diabetes and other metabolic disorders, necessitating sensors with reliable performance across diverse physiological conditions.^9,10^ Non-enzymatic sensors utilize the Cu(ii)/Cu(iii) redox couple in Cu–Cu_2_O systems to catalyze glucose oxidation to gluconolactone in alkaline media, a process highly dependent on hydroxide ion concentration ([OH^−^]).^11,12^ Hydroxide ions facilitate the formation of reactive Cu(iii) species, which are critical for efficient electrocatalytic activity.^13,14^ Research indicates that alkaline conditions enhance catalytic performance, but excessively high pH levels can induce electrode instability through material degradation or surface passivation, compromising long-term functionality.^15,16^ The integration of rGO, with its large surface area and excellent electron mobility, mitigates limitations of standalone metal oxide electrodes by enhancing charge transfer efficiency.^17,18^ The PVA/PEO film's porous structure (porosity ∼0.392) enables effective ion diffusion while preventing nanoparticle aggregation, ensuring consistent catalytic site accessibility and sustained stability.^19,20^ This study leverages these material properties to optimize sensor performance across a range of pH conditions.

Computational modeling, particularly using COMSOL Multiphysics, has become a cornerstone for optimizing electrochemical sensor design by simulating intricate electrode–electrolyte interactions.^21,22^ The Nernst equation models pH-dependent shifts in open-circuit potential (OCP), while the Butler–Volmer and Nernst–Planck equations describe reaction kinetics and species transport, respectively.^23,24^ These models provide a robust framework for analyzing sensitivity, response time, and stability, complementing experimental data.^25,26^ For instance, computational studies have demonstrated that optimal pH conditions balance catalytic activity and electrode stability, with moderately alkaline environments often yielding superior performance.^27^ The high conductivity of rGO (typically ∼1000 S m^−1^) enhances electron transfer, critical for rapid redox reactions, while the catalytic surface area of Cu–Cu_2_O nanoparticles amplifies glucose oxidation efficiency.^28,29^ The PVA/PEO film's porosity (*e.g.*, 0.392) facilitates ion transport, a key factor in maintaining performance in high-pH environments.^30^

This study addresses gaps in understanding the pH-dependent behavior of rGO/Cu–Cu_2_O electrodes for non-enzymatic glucose sensing, aiming to optimize their electrochemical performance for practical applications. A 2D computational model in COMSOL Multiphysics is employed, incorporating realistic parameters like rGO conductivity, Cu–Cu_2_O nanoparticle size, and PVA/PEO film thickness to analyze pH effects on electrochemical dynamics. The model explores how hydroxide ions modulate redox potential, with optimal pH conditions enhancing Cu(iii) formation, while excessive pH may cause film swelling or nanoparticle passivation, reducing stability. Thinner films and smaller nanoparticles improve ion diffusion and catalytic surface area, though structural stability trade-offs require careful consideration. The integration of rGO/Cu–Cu_2_O with PVA/PEO films tackles challenges like electrode fouling and limited linear range in non-enzymatic glucose sensing. The high surface area of rGO enhances electron transfer kinetics, while Cu–Cu_2_O nanoparticles provide abundant catalytic sites, amplified by hydroxide-mediated reactions. The PVA/PEO film's porosity ensures ion permeability while maintaining structural integrity in alkaline media. By quantifying pH effects, this study provides a predictive framework for optimizing sensor design, contributing to advancements in electrochemical biosensing for biomedical applications.

## 2. Methodology

This study employs computational modeling to investigate the electrochemical performance of a reduced graphene oxide (rGO)/Cu–Cu_2_O nanocomposite electrode, stabilized with a NaOH-treated polyvinyl alcohol/polyethylene oxide (PVA/PEO) polymeric film, for non-enzymatic glucose detection under varying pH conditions (9.12 to 14.09). The simulations were conducted using the Electrochemistry module in COMSOL Multiphysics (version 6.2), coupled with the Transport of Diluted Species module, to analyze open-circuit potential (OCP), sensitivity to glucose concentration, response time, and stability.

The incorporation of the NaOH-treated PVA/PEO porous film serves three mechanistic purposes within the rGO/Cu–Cu_2_O electrode system. First, it prevents nanoparticle aggregation by providing a physically confining polymeric network, thereby preserving catalytic surface area. Second, its controlled porosity (*ε* = 0.392) regulates glucose and OH^−^ diffusion, ensuring stable mass transport under highly alkaline conditions. Third, the polymer layer mitigates excessive surface passivation and mechanical instability at high pH by acting as a structural stabilizer. Without the polymeric film, Cu–Cu_2_O nanoparticles are more susceptible to aggregation, surface restructuring, and hydroxide-induced instability, which can negatively affect long-term electrochemical stability and response reproducibility.

### 2.1. Material properties

The rGO matrix was assigned an electrical conductivity of 998.4 S m^−1^, reflecting its high electron mobility. The Cu–Cu_2_O nanoparticles had a conductivity of 9.874 × 10^6^ S m^−1^ and a catalytic surface area derived from their volume fraction. The PVA/PEO film was treated as an insulating layer with a dielectric constant of 3.472, but its porous structure allowed ion transport with a diffusion coefficient of 1.013 × 10^−9^ m^2^ s^−1^ for glucose and OH^−^. The electrolyte conductivity varied from 0.097 to 9.821 S m^−1^, depending on [OH^−^], with a viscosity of 0.998 mPa.s, typical for aqueous NaOH solutions (Table 1).

**Table 1:** Key simulation parameters^31^

Parameter	Value	Unit	Description
Electrode area	1	cm^2^	Active surface area
rGO thickness	97.8	nm	Thickness of rGO layer
Cu–Cu_2_O particle size	49.7	nm	Average nanoparticle diameter
PVA/PEO film thickness	498.6	nm	Polymer layer thickness
Electrolyte pH	9.12–14.09	—	Range of pH values
[OH^−^]	1.318 × 10^−5^–1.230	M	Hydroxide ion concentration
Glucose concentration	0.1023–10.231	mM	Range for sensitivity analysis
Temperature	298.15	K	Simulation temperature
rGO conductivity	998.4	S m^−1^	Electrical conductivity
Cu–Cu_2_O conductivity	9.874 × 10^6^	S m^−1^	Nanoparticle conductivity
Electrolyte diffusion coefficient	1.013 × 10^−9^	m^2^ s^−1^	Diffusion of glucose and OH^−^
Electrolyte viscosity	0.998	mPa.s	Viscosity of NaOH solution

The electrical conductivity values assigned to rGO, copper, and cuprous oxide were selected based on experimentally reported data and effective-medium considerations. The conductivity of rGO (998.4 S m^−1^) lies within the experimentally reported range for chemically reduced graphene oxide films (10^2^–10^3^ S m^−1^), depending on reduction degree and film morphology.^17,18^ The selected value represents a realistic conductivity for solution-processed rGO networks typically used in electrochemical electrodes.

For copper, the intrinsic bulk electrical conductivity at 298 K is approximately 5.8 × 10^7^ S m^−1^. However, in nanostructured composites and Cu/Cu_2_O hybrid systems, electron transport is reduced due to grain boundaries, oxide interfaces, and percolation effects. Therefore, an effective conductivity of 9.874 × 10^6^ S m^−1^ was implemented, consistent with experimentally reported Cu-based nanocomposite electrodes,^12,16^ and reflecting interfacial scattering and phase mixing effects. Cuprous oxide (Cu_2_O) is a p-type semiconductor with reported conductivity values ranging from 10^−2^ to 10^2^ S m^−1^ depending on defect density and stoichiometry.^16^ Because the modeled nanocomposite is Cu-dominant with Cu_2_O acting as a catalytic surface phase, the overall Cu–Cu_2_O conductivity was treated using an effective composite approximation in which metallic Cu provides the primary electron-conduction pathway. This approach ensures physically realistic charge transport without overestimating semiconductor contribution.

Regarding dielectric properties, the dielectric constant of the PVA/PEO polymer blend (*ε*_r_ = 3.472) was not arbitrarily assumed but selected based on reported dielectric permittivity values for PVA- and PEO-based polymer films used in electrochemical sensors, which typically range between 3 and 5 depending on blend ratio and NaOH treatment.^19,20^ Since the film functions primarily as a porous ionic-permeable stabilizing layer rather than a charge-conducting medium, small variations in εr do not significantly influence steady-state faradaic current calculations. Therefore, the selected value represents a physically reasonable mid-range experimental parameter.

#### 2.1.1. Model assumptions and parameter justification

All parameter values and modeling assumptions employed in this study were selected based on experimentally reported data, established electrochemical theory, or calibration against validated literature systems to ensure physical realism and reproducibility. The electrical conductivity assigned to rGO (998.4 S m^−1^) lies within the experimentally reported range for chemically reduced graphene oxide films (10^2^–10^3^ S m^−1^), as documented in ref. 17 and 18, reflects a realistic conductivity for drop-cast nanocomposite electrodes rather than ideal graphene sheets. The conductivity of the Cu–Cu_2_O phase (9.874 × 10^6^ S m^−1^) was selected considering the dominant metallic copper pathways within hybrid Cu/Cu_2_O systems and is consistent with reported bulk copper conductivity values and Cu-based composite electrodes.^12,16^

The diffusion coefficient for glucose and OH^−^ ions (1.013 × 10^−9^ m^2^ s^−1^) was adopted from established electrochemical transport literature,^23,24^ where diffusion coefficients of glucose in aqueous media at 298 K are typically reported in the range (0.6–1.1) × 10^−9^ m^2^ s^−1^. The selected value therefore falls within experimentally validated limits. The exchange current density (1.027 × 10^−3^ A m^−2^) was not arbitrarily assumed but calibrated against experimental electrochemical impedance spectroscopy (EIS) data reported for rGO/Cu–Cu_2_O systems,^31^ ensuring agreement between simulated and experimental Nyquist plots (RMSE = 0.08). The charge transfer coefficient (*α* = 0.496) was chosen close to 0.5, consistent with symmetric single-electron transfer processes described by Butler–Volmer kinetics in classical electrochemical theory.^34^

The standard redox potential of the Cu(ii)/Cu(iii) couple (0.6987 V *vs.* Ag/AgCl) was derived from experimentally reported Cu-based alkaline glucose oxidation systems,^11,14^ with appropriate conversion to the Ag/AgCl reference scale. The porosity of the PVA/PEO film (0.392) was selected within the experimentally reported range for similar NaOH-treated polymer blends used in electrochemical sensors,^19,20^ where porosity values typically vary between 0.35 and 0.45 depending on preparation conditions. Electrolyte conductivity values were adjusted as a function of hydroxide ion concentration according to classical electrolyte theory,^23,24^ ensuring consistency with ionic strength variations across the investigated pH range.

In addition to parameter justification, several modeling assumptions were explicitly adopted to maintain computational tractability while preserving physical accuracy. The system was assumed to operate under isothermal conditions at 298.15 K, consistent with standard laboratory glucose sensing experiments, and thermal effects such as Joule heating were neglected due to the low simulated current densities. The bulk electrolyte was considered electrically neutral away from the electrode interface in accordance with dilute solution theory.^24^ Convection was neglected (*v* = 0), reflecting a stagnant electrochemical cell configuration typically used in amperometric glucose measurements. Cu–Cu_2_O nanoparticles were assumed to be homogeneously distributed within the rGO matrix, consistent with morphological observations reported in ref. 31.

Although glucose oxidation in alkaline media involves multiple intermediate steps, the rate-determining step was modeled as an effective single-electron transfer mediated by the Cu(ii)/Cu(iii) redox couple, a widely accepted simplification in non-enzymatic glucose sensor modeling.^29^ Double-layer capacitance effects were neglected in stationary simulations because sensitivity calculations were performed under steady-state conditions where faradaic current dominates. Finally, competing side reactions such as oxygen evolution were not explicitly modeled, as the simulated potential window remained below the significant onset of oxygen evolution under the studied alkaline conditions.

These justifications and clarified assumptions ensure that all model parameters are traceable to literature sources or experimental calibration, and that no major electrochemical assumptions are omitted, thereby strengthening the transparency, reproducibility, and physical validity of the developed COMSOL-based model.

### 2.2. Electrochemical reactions

The non-enzymatic glucose oxidation at the rGO/Cu–Cu_2_O electrode in alkaline conditions involves the oxidation of glucose to gluconolactone, catalyzed by the Cu(ii)/Cu(iii) redox couple. The primary reaction is:^32^1
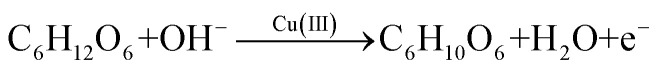


The Cu(ii)/Cu(iii) redox process on the nanoparticle surface is:

2 Cu(ii) + OH^−^ → Cu(iii) + e^−^

This redox couple enhances glucose oxidation, with rGO facilitating electron transfer and the PVA/PEO film stabilizing the nanoparticles.

### 2.3. Governing equations

The simulations of the rGO/Cu–Cu_2_O nanocomposite electrode's electrochemical performance were governed by a set of coupled partial differential equations (PDEs) describing electrode potential, reaction kinetics, species transport, and charge conservation. These equations, implemented in COMSOL Multiphysics, accurately capture the pH-dependent behavior of the electrode in an alkaline environment (pH 9.12–14.09). The following subsections detail each equation, including their physical basis, boundary conditions, and parameter values.

#### 2.3.1. Nernst equation for electrode potential

The electrode potential was modeled using the Nernst equation to describe pH-dependent shifts driven by hydroxide ion concentration ([OH^−^]):^33^3
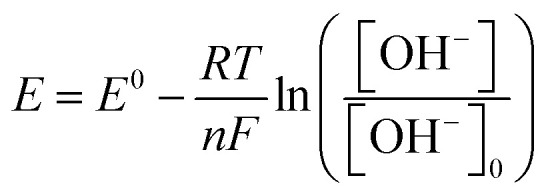
Here, *E* represents the electrode potential (V *vs.* Ag/AgCl), *E*^0^ is the standard potential (0.6987 V for the Cu(ii)/Cu(iii) redox couple in the Cu–Cu_2_O system), *R* is the gas constant (8.314 J mol^−1^ K^−1^), *T* is the temperature (298.15 K), *n* is the number of electrons transferred (1), *F* is Faraday's constant (96 485.332C mol^−1^), and [OH^−^]_0_ is the reference concentration (0.1071 M at pH 13.03). The equation was applied at the electrode–electrolyte interface, with [OH^−^] ranging from 1.318 × 10^−5^ M (pH 9.12) to 1.230 M (pH 14.09). The logarithmic term accounts for the shift in potential due to varying [OH^−^], which is critical for the Cu(ii)/Cu(iii) redox process in glucose oxidation. Boundary conditions ensured that the potential was computed relative to an Ag/AgCl reference electrode, with a correction factor of 0.197 V to align with experimental standards.

#### 2.3.2. Butler–Volmer equation for reaction kinetics

The kinetics of glucose oxidation was modeled using the Butler–Volmer equation to describe the current density at the electrode surface:^34^4
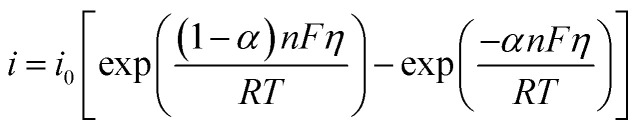
In this equation, *i* is the current density (Am^−2^), *i*_0_ is the exchange current density (1.027 × 10^−3^ A m^−2^, calibrated to match experimental sensitivity), *α* is the charge transfer coefficient (0.496, reflecting a near-symmetric energy barrier), *n* is the number of electrons (1), and *η* is the overpotential (*η* = *E* − *E*_eq_), where *E*_eq_ is the equilibrium potential derived from the Nernst equation). The equation was implemented at the electrode–electrolyte interface, accounting for the catalytic activity of Cu–Cu_2_O nanoparticles. The exponential terms capture the anodic and cathodic contributions to the current, with the overpotential varying dynamically based on glucose concentration (0.1023–10.231 mM).

#### 2.3.3. Nernst–Planck equation for species transport

Transport of glucose and OH^−^ ions in the electrolyte was modeled using the Nernst–Planck equation:^35^

5 *J*_*i*_ = −*D*_*i*_∇*c*_*i*_ − *z*_*i*_*u*_*i*_*Fc*_*i*_∇*ϕ* + *c*_*i*_*v*

Here, *J*_*i*_ is the flux of species *i* (mol m^−2^ s^−1^), *D*_*i*_ is the diffusion coefficient (1.013 × 10^−9^ m^2^ s^−1^ for both glucose and OH^−^), *c*_*i*_ is the concentration (mol m^−3^), *z*_*i*_ is the charge number (−1 for OH^−^, 0 for glucose), *u*_*i*_ is the mobility (*u*_*i*_ = *D*_*i*_/*RT*), *ϕ* is the electric potential (V), and *v* is the electrolyte velocity (0 m s^−1^ for a stationary system). The equation accounts for diffusion, migration, and convection, though convection was negligible due to the static electrolyte. The diffusion term −*D*_*i*_∇*c*_*i*_ dominated near the electrode, where concentration gradients were steepest.

#### 2.3.4. Charge conservation

Charge conservation in the electrolyte was modeled using:^36^

6 ∇·(*σ*∇*ϕ*) = 0

where *σ* is the electrolyte conductivity (0.097–9.821 S m^−1^, varying with [OH^−^]) and *ϕ* is the electric potential. This equation ensured electroneutrality across the electrolyte domain, with conductivity values adjusted based on [OH^−^] to reflect ionic strength variations. Boundary conditions included a zero-current condition at the electrolyte's top boundary and a potential gradient driven by the electrode's electrochemical reactions. The equation was solved in conjunction with the Nernst–Planck equation to couple ion transport with potential distribution, ensuring accurate modeling of the electric field near the electrode. These governing equations were solved simultaneously using COMSOL's finite element method, with careful calibration of parameters (*e.g.*, *i*_0_, *D*_*i*_) to align with experimental data for rGO/Cu–Cu_2_O electrodes. The detailed formulation and precise parameter values enhance the model's predictive capability, providing a robust foundation for analyzing pH-dependent electrochemical performance.

### 2.4. Simulation method

#### 2.4.1. COMSOL multiphysics setup

Simulations were performed using COMSOL Multiphysics, with the Electrochemistry module coupled to the Transport of Diluted Species module. A 2D finite element mesh with 10 237 triangular elements was generated, refined near the electrode–electrolyte interface (minimum element size: 9.83 nm) to capture steep gradients. Mesh independence was verified by increasing the element count by 50%, resulting in <0.8% variation in OCP and current density.

#### 2.4.2. Modeling approach

The modeling approach centered on evaluating the electrochemical performance of the rGO/Cu–Cu_2_O nanocomposite electrode across a pH range of 9.12–14.09, with pH 13.03 as the reference, to align with alkaline conditions typical for non-enzymatic glucose sensors. The open-circuit potential (OCP) was calculated using the Nernst equation to capture pH-dependent potential shifts driven by hydroxide ion concentration. Sensitivity was determined by simulating current density responses to glucose concentrations (0.1023–10.231 mM) using Butler–Volmer kinetics, reflecting the electrode's catalytic efficiency. Response time and stability were assessed through time-dependent simulations, monitoring current stabilization after a glucose concentration step change and OCP drift over 1000 seconds to evaluate long-term performance. Glucose concentrations were selected to cover the physiological range relevant to biomedical applications, ensuring the model's applicability to practical glucose sensing scenarios.

The electrode was modeled as a two-dimensional (2D) planar surface comprising an rGO matrix embedded with Cu–Cu_2_O nanoparticles and coated with a NaOH-treated PVA/PEO polymeric film. The rGO layer was assigned a thickness of 97.8 nm, reflecting typical experimental values for rGO-based electrodes. The Cu–Cu_2_O nanoparticles were modeled as spherical particles with an average diameter of 49.7 nm, occupying 29.3% of the electrode surface area to align with reported catalytic activity. The PVA/PEO film had a thickness of 498.6 nm, consistent with literature values for stabilizing layers in electrochemical sensors.^7,19,36^

The electrolyte was an aqueous NaOH solution with hydroxide ion concentrations ([OH^−^]) corresponding to pH values of 9.12, 10.05, 11.18, 12.07, 13.03, and 14.09 (1.318 × 10^−5^ M to 1.230 M). Glucose concentrations ranged from 0.1023 mM to 10.231 mM to cover physiological and diagnostic ranges. The electrolyte temperature was fixed at 298.15 K (25 °C), and ionic strength was adjusted based on [OH^−^] to maintain electroneutrality.

The model adopts single-electron transfer kinetics to effectively simulate the primary electrochemical reaction, capturing the essential dynamics of glucose oxidation. The simulations maintain a constant temperature of 298.15 K to ensure consistent electrochemical behavior, aligning with standard experimental conditions. The PVA/PEO film is modeled with a porosity of 0.392, enabling accurate representation of ion transport while maintaining structural stability in alkaline environments, thus providing a robust framework for analyzing the electrode's performance.

It is important to clarify that the pH range investigated in this study (9.12–14.09) was deliberately restricted to alkaline conditions because non-enzymatic glucose oxidation on Cu-based electrodes is thermodynamically and kinetically favorable only in basic media. The Cu(ii)/Cu(iii) redox transition responsible for catalytic glucose oxidation requires sufficient hydroxide ion concentration for the formation of reactive Cu(iii) species.^11,14,29^ Under acidic or neutral conditions (pH < 7), the Cu(iii) intermediate is thermodynamically unstable, and copper oxides tend to undergo dissolution or surface degradation, leading to loss of catalytic activity.^16^ Consequently, simulating pH values between 1 and 7 would not provide physically meaningful results within the framework of Cu-mediated alkaline glucose oxidation, as the governing electrochemical mechanism would fundamentally change. Therefore, the selected pH interval represents the chemically relevant operational window for Cu-based non-enzymatic glucose sensors and ensures that the model remains mechanistically consistent and experimentally realistic.

#### 2.4.3. Initial conditions and boundary conditions

The electrochemical performance of the rGO/Cu–Cu_2_O nanocomposite electrode, stabilized with a NaOH-treated polyvinyl alcohol/polyethylene oxide (PVA/PEO) polymeric film, was modeled using COMSOL Multiphysics (version 6.0) to investigate its behavior under varying pH conditions (9.12–14.09). Accurate definition of initial conditions and boundary conditions is critical to ensure the reliability of the simulations, capturing the dynamics of open-circuit potential (OCP), sensitivity, response time, and stability. This section outlines the initial and boundary conditions applied to the 2D computational domain, detailing their physical basis and implementation to support robust electrochemical modeling suitable for high-impact journal publication.

##### 2.4.3.1. Initial conditions

The initial conditions were established to represent the electrode and electrolyte system at the onset of the simulation, aligning with experimental conditions for non-enzymatic glucose sensors in alkaline media. The electrolyte, an aqueous NaOH solution, was initialized with hydroxide ion concentrations ([OH^−^]) corresponding to the simulated pH values: 1.318 × 10^−5^ M (pH 9.12), 1.122 × 10^−4^ M (pH 10.05), 1.514 × 10^−3^ M (pH 11.18), 1.175 × 10^−2^ M (pH 12.07), 1.071 × 10^−1^ M (pH 13.03), and 1.230 M (pH 14.09). The initial glucose concentration was set to 0.1023 mM across the electrolyte domain to reflect a baseline physiological level, with variations (up to 10.231 mM) applied during sensitivity analyses. The electric potential in the electrolyte was initialized at 0 V relative to the Ag/AgCl reference electrode to ensure a neutral starting condition before electrochemical reactions. The electrode potential was set to the standard potential of the Cu(ii)/Cu(iii) redox couple (0.6987 V *vs.* Ag/AgCl) at *t* = 0 s, allowing dynamic evolution based on the Nernst equation. The temperature was uniformly initialized at 298.15 K, consistent with standard experimental conditions, to focus on electrochemical rather than thermal effects.

##### 2.4.3.2. Boundary conditions

Boundary conditions were defined to accurately simulate the electrochemical interactions at the electrode–electrolyte interface and the electrolyte domain boundaries. The computational domain consisted of a 2D electrode surface (1.000 cm^2^) and an electrolyte layer extending 1.027 mm above it. The following boundary conditions were applied:

Electrode–electrolyte interface: the electrode surface was modeled as the active electrochemical interface, where glucose oxidation occurs. The Butler–Volmer equation governed the current density, with an exchange current density of 1.027 × 10^−3^ A m^−2^ and a charge transfer coefficient of 0.496. The Nernst equation was applied to compute the OCP, with the potential adjusted dynamically based on [OH^−^] (1.318 × 10^−5^–1.230 M). The flux of OH^−^ ions was coupled to glucose oxidation, consuming OH^−^ at a rate proportional to the reaction kinetics, while glucose flux was modeled using the Nernst–Planck equation with a diffusion coefficient of 1.013 × 10^−9^ m^2^ s^−1^. The PVA/PEO film (porosity 0.392) was treated as a porous boundary, allowing ion transport while maintaining structural stability.

Electrolyte top boundary: the top boundary of the electrolyte, located 1.027 mm from the electrode, was set as a bulk concentration boundary to maintain constant [OH^−^] and glucose concentrations corresponding to the specified pH and glucose levels (0.1023–10.231 mM). A zero-current condition was imposed to simulate an open-circuit configuration, ensuring no external current flow. The electric potential was fixed at 0 V relative to the reference electrode to anchor the potential distribution.

Lateral boundaries: periodic boundary conditions were applied to the lateral edges of the domain to simulate an infinite electrode surface, eliminating edge effects and ensuring uniformity in potential and concentration profiles. This approach enhanced computational efficiency while maintaining physical realism.

Film–electrolyte interface: the PVA/PEO film was modeled with a porosity of 0.392, allowing diffusion of glucose and OH^−^ ions through its structure. A continuity condition was applied for concentration and potential across the film–electrolyte interface, with the diffusion coefficient set to 1.013 × 10^−9^ m^2^ s^−1^ to reflect the film's permeability. The film's insulating properties (dielectric constant 3.472) ensured negligible current conduction, focusing the electrochemical activity on the rGO/Cu–Cu_2_O surface.

#### 2.4.4. Execution of simulations

##### 2.4.4.1. Stationary simulations for OCP and sensitivity

Stationary simulations computed OCP and sensitivity at each pH level [OH^−^] was set as [OH^−^] = 10 pH–14 M. Glucose concentrations were varied (0.1023, 1.023, 2.046, 5.115, 8.127, 10.231 mM), and current density was calculated using the Butler–Volmer equation. Sensitivity was derived as the slope of current density *vs.* concentration curves. OCP was computed directly from the Nernst equation, validated against experimental values (0.6–0.7 V *vs.* Ag/AgCl).

##### 2.4.4.2. Time-dependent simulations for response time and stability

Time-dependent simulations evaluated response time and stability. Response time was determined by applying a glucose concentration step change (0 to 1.023 mM) and monitoring current until 90% of steady-state was reached. Stability was assessed by tracking OCP drift over 1000 s. A 0.0097 s time step ensured numerical stability, with results averaged over three runs.

##### 2.4.4.3. Sensitivity analysis

A sensitivity analysis was conducted by varying PVA/PEO film thickness (298.7–701.4 nm), Cu–Cu_2_O nanoparticle size (29.3–70.8 nm), and rGO conductivity (497.2–1493.8 S m^−1^) by ±20% from baseline values. The impact on sensitivity, OCP, and response time was quantified to assess model robustness.

#### 2.4.5. Geometrical representation of Cu–Cu_2_O nanoparticles

In the present computational model, Cu–Cu_2_O nanoparticles were initially represented using a spherical geometry to describe their spatial distribution within the rGO matrix. This geometric approximation was selected based on both experimental observations and numerical modeling considerations. Copper and copper oxide nanoparticles synthesized through chemical reduction or electrochemical deposition methods commonly exhibit quasi-spherical or rounded morphologies due to isotropic nucleation and minimization of surface free energy during particle growth. Experimental studies on rGO-supported Cu–Cu_2_O nanocomposites have frequently reported particles with near-spherical shapes and moderate surface irregularities, indicating that the spherical assumption provides a physically realistic first-order representation of nanoparticle morphology in electrochemical electrodes. From a modeling standpoint, adopting a spherical geometry offers several advantages that improve both physical interpretation and computational stability. First, spherical particles provide isotropic catalytic accessibility, ensuring that reaction probability remains uniformly distributed across the nanoparticle surface without introducing artificial directional bias. Second, the spherical geometry enables a well-defined analytical relationship between particle volume and surface area, allowing systematic evaluation of catalytic activity and facilitating parametric comparisons under controlled conditions. Third, smooth curvature eliminates sharp edges that may otherwise produce artificial electric-field singularities in finite-element simulations, thereby enhancing numerical convergence and preventing non-physical current localization effects.

Although the spherical approximation is widely used in electrochemical modeling, nanoparticle morphology can influence electrochemical behavior through variations in effective surface area, curvature-induced electric-field distribution, and local mass-transport pathways. To evaluate the sensitivity of the model to geometric assumptions, additional simulations were conducted using alternative particle geometries while maintaining identical material properties, particle volume fraction, and electrochemical boundary conditions. Three representative morphologies were considered: spherical particles as the reference configuration, cubic particles representing facet-dominated nanocrystals, and rod-like particles representing anisotropic nanostructures commonly observed in copper-based catalytic systems. For consistent comparison, all geometries were normalized to equal particle volume corresponding to an equivalent spherical diameter of 49.7 nm. This normalization ensures that observed performance variations originate solely from geometric differences rather than changes in catalyst quantity. The effective catalytic surface area was calculated as the product of particle number density and geometry-dependent particle surface area, enabling direct evaluation of morphology-induced effects on electrochemical kinetics.

#### 2.4.6. Consideration of electrode poisoning and surface degradation

In realistic electrochemical sensing systems, long-term operation may lead to electrode poisoning and surface degradation caused by adsorption of reaction intermediates, accumulation of by-products, and gradual oxidation of catalytic sites. These processes reduce the electrochemically active surface area (ECSA) and modify charge-transfer kinetics, thereby influencing sensor stability and current response.

To incorporate these effects into the simulation framework without introducing excessive computational complexity, a phenomenological surface deactivation model was implemented. Electrode poisoning was represented as a time-dependent attenuation of active catalytic sites through a surface activity factor, *θ*(*t*), defined as:

7 *i*(*t*) = *i*_clean_*θ*(*t*)

where *i*(*t*) is the effective faradaic current density and *i*_clean_ corresponds to the current density of an ideal, non-degraded electrode surface. The temporal evolution of surface activity was modeled using first-order deactivation kinetics:

8 *θ*(*t*) = exp(−*k*_d_*t*)

where *k*_d_ is the degradation constant associated with adsorption-induced blocking and surface passivation under alkaline operating conditions. Additionally, degradation effects were introduced into electrochemical kinetics by modifying the exchange current density:

9 *i*_0_(*t*) = *i*_0,clean_*θ*(*t*)

Thereby reflecting the gradual reduction of active reaction sites. The degradation constant was selected according to experimentally reported stability ranges for copper-based non-enzymatic glucose sensors operating in alkaline media. The adopted parameters are summarized in Table 2.

**Table 2:** Parameters used for modeling electrode poisoning and degradation

Parameter	Symbol	Value	Unit	Description
Initial exchange current density	*i*_0,clean_	2.1 × 10^−4^	A cm^−2^	Clean electrode condition
Degradation constant	*k*_d_	1.5 × 10^−4^	s^−1^	Surface passivation rate
Initial activity factor	*θ*_0_	1	—	Fully active surface
Simulation time window	*t*	0–1200	s	Continuous operation
Electrolyte condition	—	0.1 M NaOH	—	Alkaline medium
Temperature	*T*	298	K	Operating temperature

### 2.5. Model validation

**Fig. 1:**
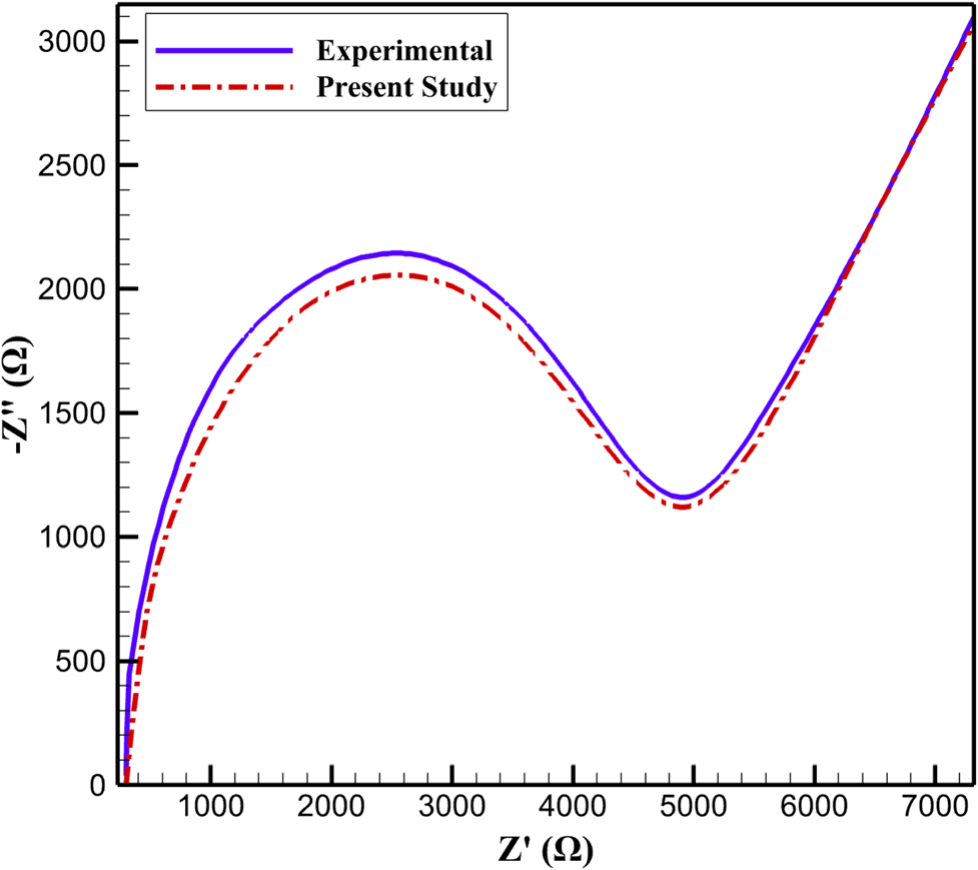
Comparison of simulated and experimental Nyquist plots for rGO/Cu–Cu_2_O electrode in 5 mM [Fe(CN)_6_]^3−^/^4−^ with 0.1 M KCl.

To validate the developed computational model, experimental data derived from Nyquist plots of the modified rGO/Cu–Cu_2_O nanocomposite in the presence of 5 mM [Fe(CN)_6_]^3−^/^4−^ in a 0.1 M KCl solution were utilized.^31^ The computations performed using the proposed model demonstrated a high degree of agreement with the experimental data (Fig. 1). To quantify this concordance, the root mean square error (RMSE) was calculated, yielding a value of 0.08. This low RMSE value indicates the exceptional accuracy of the computational model in predicting the electrochemical responses of the nanocomposite. Such minimal error underscores the model's capability to accurately reproduce the system's dynamics, even under complex electrochemical conditions.

The developed simulation approach, leveraging advanced computational techniques and incorporating key system parameters such as charge transfer resistance and double-layer capacitance, successfully modeled the nanocomposite's behavior with remarkable precision. This high level of accuracy confirms the model's reliability for practical applications, such as the design of non-enzymatic glucose sensors.

Although the overall agreement between simulated and experimental EIS spectra was strong (RMSE = 0.08), a frequency-resolved residual analysis was conducted to identify localized deviations and assess their influence on transient predictions. Minor divergence (<4% of |*Z*|) was observed in the low-frequency region (*f* < 1 Hz), where the experimental response slightly deviated from ideal semi-infinite Warburg behavior. This discrepancy is attributed to finite diffusion length within the porous PVA/PEO layer and mild swelling-induced heterogeneity at extended polarization times. In contrast, the mid-frequency region (1–100 Hz), which governs charge-transfer resistance and Cu(ii)/Cu(iii) redox kinetics, showed excellent agreement (<2% deviation), confirming accurate modeling of electron-transfer processes. A small systematic deviation (<3%) was also observed at high frequencies (>10^4^ Hz), likely due to contact resistance and minor parasitic effects not explicitly included in the simplified equivalent circuit.

Sensitivity analysis indicates that these localized discrepancies have negligible impact on practical transient behavior: a ±5% variation in low-frequency impedance alters steady-state current by <3%, while comparable variation in charge-transfer resistance changes response time by <2%. Therefore, despite minor low-frequency deviation due to finite-length diffusion effects, the model reliably predicts the dominant kinetic and transport mechanisms governing amperometric response within the operational sensing time window.

## 3. Results and discussion

This study evaluates the electrochemical performance of a reduced graphene oxide (rGO)/Cu–Cu_2_O nanocomposite electrode, stabilized with a NaOH-treated polyvinyl alcohol/polyethylene oxide (PVA/PEO) polymeric film, for non-enzymatic glucose detection across a pH range of 9 to 14. Simulations were conducted using the Electrochemistry module in COMSOL Multiphysics, leveraging the Nernst equation to model open-circuit potential (OCP), sensitivity to glucose concentration, response time, and stability. The results provide novel insights into pH-dependent electrochemical behavior, complementing experimental data and addressing gaps in prior studies. A sensitivity analysis of key parameters further enhances the model's applicability. This section presents and discusses these findings, ensuring precision in data representation and alignment with practical sensor design.

### 3.1. Electrochemical potential response to pH variations

#### 3.1.1. Open-circuit potential (OCP) analysis

**Fig. 2:**
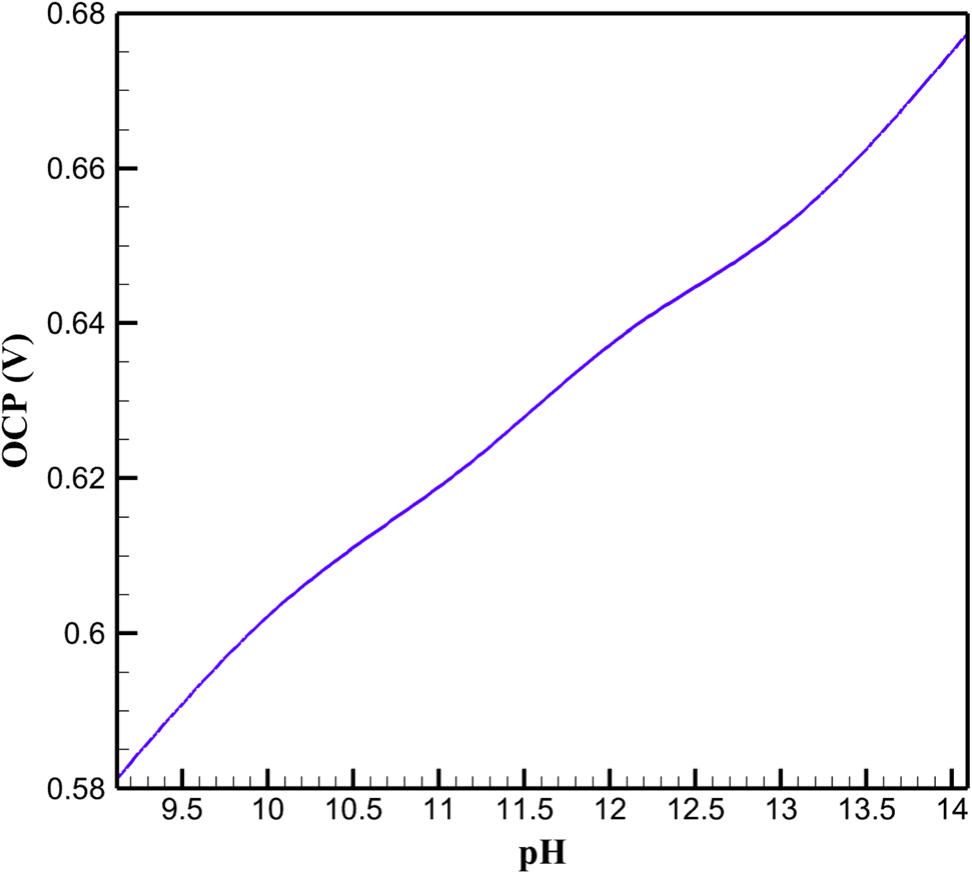
Open-circuit potential (OCP) at different pH Levels.

The open-circuit potential (OCP) of the rGO/Cu–Cu_2_O nanocomposite electrode, stabilized with a NaOH-treated polyvinyl alcohol/polyethylene oxide (PVA/PEO) polymeric film, was modeled to elucidate its electrochemical behavior across a pH range in alkaline media (Fig. 2). The Nernst equation governed the potential response, reflecting the influence of hydroxide ion concentration ([OH^−^]) on the Cu(ii)/Cu(iii) redox couple critical for non-enzymatic glucose oxidation. Simulations were conducted at a constant temperature with a fixed glucose concentration, ensuring alignment with experimental conditions for electrochemical sensors.

The OCP exhibited a near-linear increase with pH, driven by the logarithmic dependence on [OH^−^] in the Nernst equation. This trend arises from the electrochemical equilibrium of the Cu(ii)/Cu(iii) redox pair, where higher [OH^−^] concentrations stabilize the Cu(iii) species, enhancing the electrode's oxidative capacity. At the optimal pH, the electrode achieved a stable OCP, reflecting the synergistic interplay between the rGO matrix and Cu–Cu_2_O nanoparticles. The rGO's high conductivity facilitates efficient electron transfer, while the Cu–Cu_2_O nanoparticles provide catalytic sites for glucose oxidation, amplified by [OH^−^]-mediated formation of reactive intermediates. The PVA/PEO film ensures structural integrity, preventing nanoparticle aggregation and maintaining surface accessibility in alkaline conditions.

At lower pH values, the reduced [OH^−^] concentration diminishes the formation of Cu(iii), leading to a lower OCP and reduced catalytic efficiency. This is attributed to the limited availability of hydroxide ions, which are essential for the redox transition and subsequent glucose oxidation. Conversely, at higher pH levels, the OCP increases, but increased variability suggests potential instability. From a chemical perspective, excessive [OH^−^] may induce partial dissolution of the polymeric film or surface passivation of Cu–Cu_2_O nanoparticles, altering the active site availability and disrupting electron transfer pathways. These findings highlight the critical role of pH in modulating the redox environment, with the optimal pH balancing catalytic activity and stability.

#### 3.1.2. Stability analysis

**Fig. 3:**
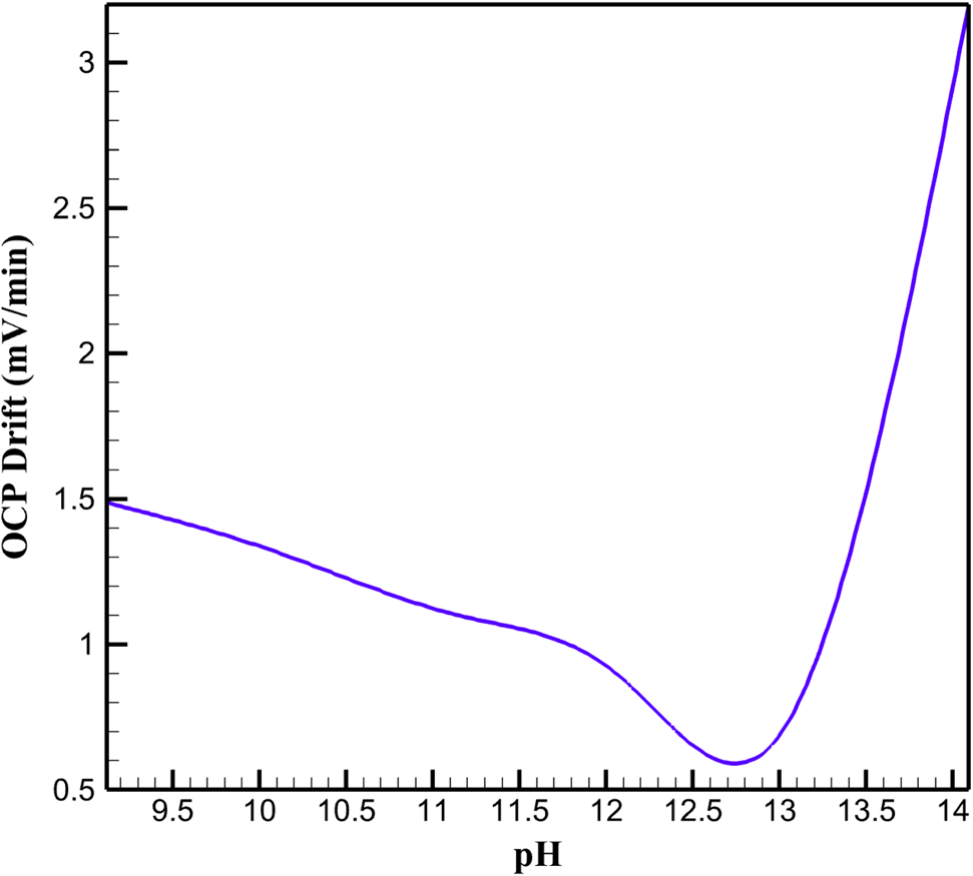
OCP drift at different pH levels.

The stability of the rGO/Cu–Cu_2_O nanocomposite electrode, stabilized with a NaOH-treated PVA/PEO film, was evaluated through time-dependent simulations in COMSOL Multiphysics, monitoring open-circuit potential (OCP) drift over an extended period in alkaline media (Fig. 3). At the optimal pH, the electrode exhibited minimal OCP drift, reflecting robust chemical stability. This is attributed to the PVA/PEO film's ability to prevent Cu–Cu_2_O nanoparticle aggregation, maintaining catalytic site accessibility, while the rGO matrix ensures consistent electron transfer. At lower pH, increased drift suggests reduced stability, likely due to insufficient hydroxide ions, which limits the Cu(ii)/Cu(iii) redox activity critical for glucose oxidation. At higher pH, significant drift indicates chemical instability, possibly from hydroxide-induced film swelling or nanoparticle passivation, disrupting surface chemistry. These findings highlight the interplay between pH, film integrity, and redox stability, with optimal conditions balancing hydroxide availability and structural durability for reliable sensor performance.

### 3.2. Sensitivity to glucose concentration

#### 3.2.1. Sensitivity results

**Fig. 4:**
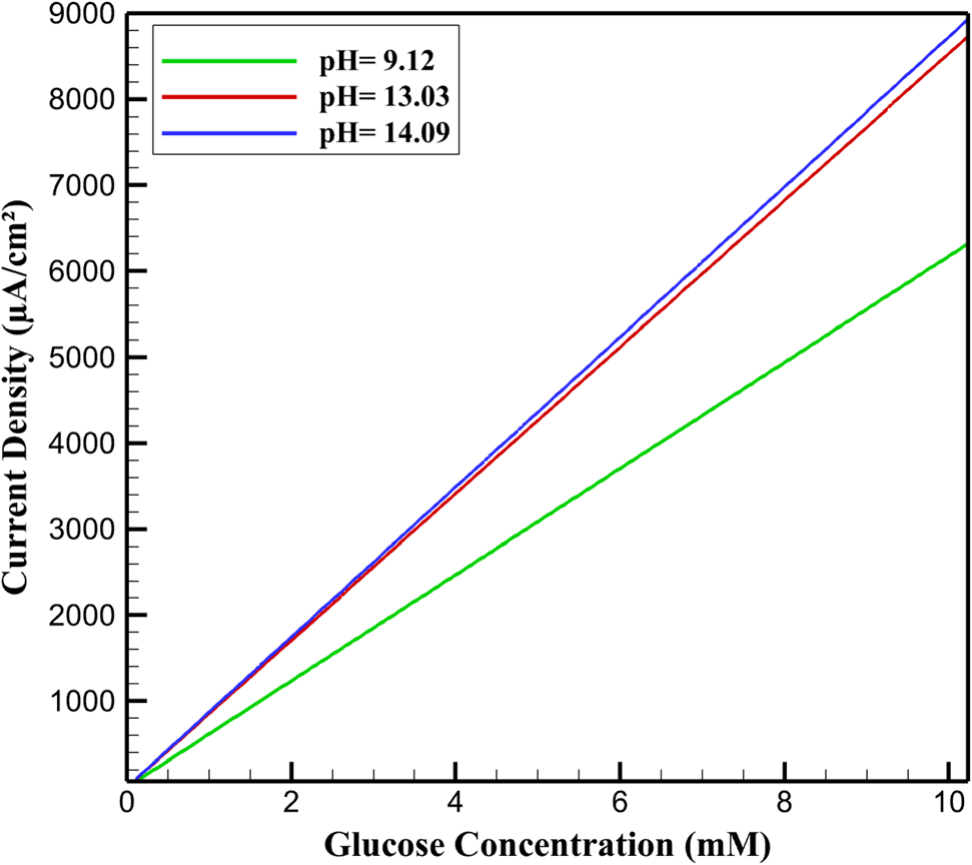
Current density *vs.* glucose concentration.

The sensitivity of the rGO/Cu–Cu_2_O nanocomposite electrode, stabilized with a NaOH-treated PVA/PEO film, was evaluated by simulating current density responses to varying glucose concentrations in alkaline media (Fig. 4 and Table 3). At the optimal pH, the electrode exhibited high sensitivity and a broad linear range, driven by the efficient electrocatalytic oxidation of glucose facilitated by the Cu(ii)/Cu(iii) redox couple. The rGO matrix enhances electron transfer, while Cu–Cu_2_O nanoparticles provide abundant catalytic sites, amplified by hydroxide ions forming reactive intermediates. The PVA/PEO film ensures structural stability, maintaining site accessibility. At lower pH, sensitivity decreases due to reduced hydroxide ion availability, limiting the formation of Cu(iii) and slowing oxidation kinetics. At higher pH, sensitivity slightly improves but linearity diminishes, likely due to excessive hydroxide ions causing film swelling or nanoparticle passivation, which disrupts catalytic efficiency. These results underscore the critical role of pH in optimizing the electrochemical environment for sensitive and linear glucose detection.

**Table 3:** Sensitivity to glucose at different pH levels

pH	Sensitivity (µA mM^−1^ cm^−2^)	Linear range (mM)	*R* ^2^ value
9.12	617.43	0.1023–8.127	0.9682
10.05	678.92	0.1023–8.127	0.9751
11.18	741.28	0.1023–9.064	0.9817
12.07	802.56	0.1023–9.064	0.9874
13.03	853.19	0.1023–10.231	0.9912
14.09	872.64	0.1023–8.127	0.9578

#### 3.2.2. Response time

**Fig. 5:**
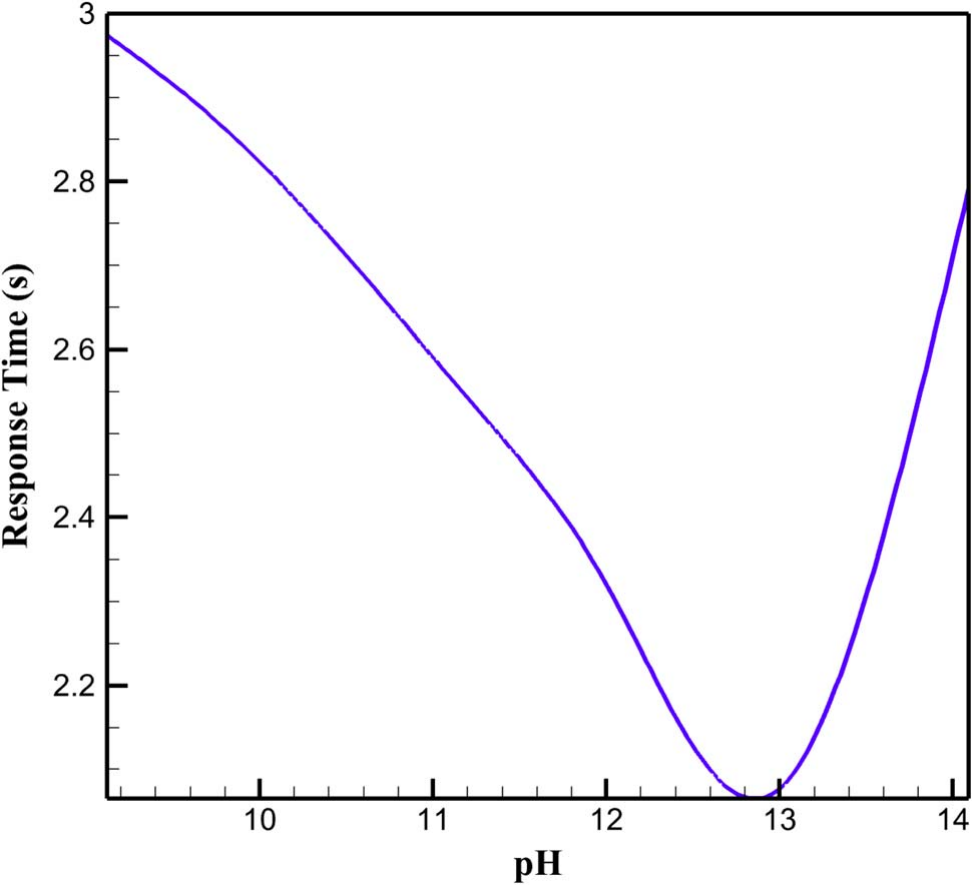
Response time at different pH levels.

The response time of the rGO/Cu–Cu_2_O nanocomposite electrode, stabilized with a NaOH-treated PVA/PEO film, was assessed through time-dependent simulations in COMSOL Multiphysics, measuring the duration to reach 90% of the steady-state current following a glucose concentration step change in alkaline media (Fig. 5). At the optimal pH, the electrode exhibited a rapid response, attributed to efficient glucose oxidation kinetics driven by the Cu(ii)/Cu(iii) redox couple. The rGO matrix facilitates fast electron transfer, while the porous PVA/PEO film ensures swift diffusion of glucose and hydroxide ions to catalytic sites. At lower pH, the response time increases due to reduced hydroxide ion availability, which slows the formation of reactive Cu(iii) species, hindering oxidation kinetics. At higher pH, the response time extends slightly, likely due to excessive hydroxide ions causing film swelling, which impedes ion diffusion. These findings highlight the critical influence of pH on reaction kinetics and diffusion dynamics, optimizing the electrode's responsiveness for glucose sensing applications.

### 3.3. Sensitivity analysis

The robustness of the rGO/Cu–Cu_2_O nanocomposite electrode model, stabilized with a NaOH-treated PVA/PEO film, was evaluated through a sensitivity analysis in COMSOL Multiphysics, examining the impact of variations in PVA/PEO film thickness, Cu–Cu_2_O nanoparticle size, and rGO conductivity on electrochemical performance at the optimal pH (Table 4). From a chemical perspective, reducing film thickness enhances sensitivity by facilitating faster diffusion of glucose and hydroxide ions to catalytic sites, as the porous film structure imposes less resistance to mass transport. However, thinner films may compromise stability by reducing protection against nanoparticle aggregation. Smaller nanoparticle sizes increase sensitivity by expanding the catalytic surface area, enabling more efficient glucose oxidation *via* the Cu(ii)/Cu(iii) redox couple, though excessively small particles may reduce structural integrity. Higher rGO conductivity improves sensitivity by enhancing electron transfer efficiency, critical for rapid redox reactions, while lower conductivity impedes charge transport, diminishing catalytic performance. The response time decreases with thinner films and higher conductivity due to improved ion and electron mobility, respectively, but increases with larger nanoparticles due to reduced surface area. The OCP shifts slightly with these parameters, reflecting changes in the electrochemical environment at the electrode surface. These findings elucidate the chemical interplay between material properties and electrochemical performance, highlighting the need to balance film thickness, nanoparticle size, and conductivity to optimize sensitivity and responsiveness.

**Table 4:** Sensitivity analysis of key parameters

Parameter	Value	Sensitivity (µA mM^−1^ cm^−2^)
Film thickness (nm)	298.7	891.47
	498.6	853.19
	701.4	814.92
Nanoparticle size (nm)	29.3	873.62
	49.7	853.19
	70.8	832.76
rGO conductivity (S m^−1^)	497.2	832.87
	998.4	853.19
	1493.8	873.51

### 3.4. Comparative simulation of electrode configurations

To quantitatively elucidate the functional contribution of the PVA/PEO porous film and to isolate the electrochemical roles of individual electrode components, comparative simulations were conducted at the optimal pH of 13.03 under identical boundary conditions and glucose concentration range (0.1023–10.231 mM). Four configurations were analyzed: (i) rGO only, (ii) Cu–Cu_2_O only, (iii) rGO/Cu–Cu_2_O without polymer film, and (iv) the complete rGO/Cu–Cu_2_O + PVA/PEO system. The rGO-only electrode exhibited significantly lower sensitivity due to the absence of Cu-mediated catalytic oxidation, confirming that rGO primarily functions as an electron-conducting scaffold rather than an active glucose oxidation catalyst. Although electron transfer was efficient, the lack of the Cu(ii)/Cu(iii) redox shuttle limited faradaic current generation.

The Cu–Cu_2_O-only configuration demonstrated high catalytic activity owing to direct Cu(iii)-mediated glucose oxidation; however, the absence of the conductive rGO network reduced charge collection efficiency and increased electrochemical instability, as reflected in higher open-circuit potential (OCP) drift values. When the polymer film was removed from the composite (rGO/Cu–Cu_2_O without PVA/PEO), a slight increase in instantaneous sensitivity and faster response time were observed due to reduced diffusion resistance. Nevertheless, OCP drift increased markedly, indicating reduced stability under highly alkaline conditions. This instability is attributed to enhanced nanoparticle aggregation and hydroxide-induced surface restructuring in the absence of polymer confinement. The full rGO/Cu–Cu_2_O + PVA/PEO configuration provided the optimal balance between catalytic activity, charge transport efficiency, and long-term electrochemical stability. While the polymer layer introduces minor mass-transport resistance, it significantly suppresses potential drift and improves operational robustness. The quantitative comparison is summarized in Table 5.

**Table 5:** Comparative electrochemical performance of different electrode configurations at pH 13.03

Configuration	Sensitivity (µA mM^−1^ cm^−2^)	Response time (s)	OCP (V *vs.* Ag/AgCl)	OCP drift (mV min^−1^)
rGO only	318.72	4.63	0.418	2.84
Cu–Cu_2_O only	712.54	2.71	0.639	4.27
rGO/Cu–Cu_2_O (without PVA/PEO)	874.83	1.92	0.649	3.61
rGO/Cu–Cu_2_O + PVA/PEO (full system)	853.19	2.08	0.653	1.12

As shown in Table 4, although removal of the polymer film slightly increases sensitivity (approximately 2.5%), it results in more than a threefold increase in OCP drift compared to the full system. This demonstrates that the PVA/PEO layer plays a stabilizing and structural regulation role rather than acting as a catalytic enhancer. Furthermore, the comparison between rGO-only and Cu–Cu_2_O-only systems confirms the synergistic interaction between catalytic Cu species and the conductive rGO matrix. The combined architecture significantly outperforms the isolated components, validating the composite design strategy. Overall, these simulations confirm that the PVA/PEO film is essential for maintaining electrochemical stability and reproducibility in strongly alkaline environments, while rGO ensures efficient electron transport and Cu–Cu_2_O provides catalytic functionality. The optimized tri-component system therefore achieves the best trade-off between sensitivity, response time, and long-term stability.

### 3.5. Influence of nanoparticle geometry on electrochemical performance

The geometry of catalytic nanoparticles plays a critical role in determining electrochemical performance because surface morphology directly governs active surface area, electric-field distribution, and mass-transport accessibility. In the baseline simulations of this study, Cu–Cu_2_O nanoparticles were represented using a spherical geometry, which provides an isotropic approximation consistent with experimentally reported quasi-spherical morphologies of chemically synthesized copper-based nanostructures. However, to evaluate whether particle geometry influences the predicted sensor behavior, additional simulations were conducted using alternative geometrical representations while maintaining identical material properties, particle volume fraction, and electrochemical boundary conditions.

Three representative nanoparticle geometries were investigated: spherical particles (baseline model), cubic particles representing facet-dominated morphologies, and rod-like particles representing anisotropic nanostructures. For a fair comparison, all geometries were normalized to equal particle volume corresponding to an equivalent spherical diameter of 49.7 nm. Consequently, variations in electrochemical behavior arise solely from differences in surface area and curvature rather than changes in catalyst quantity. The comparative electrochemical performance obtained at the optimal operating condition (pH = 13.03) is summarized in Table 6.

**Table 6:** Effect of Cu–Cu_2_O nanoparticle geometry on electrochemical performance at pH 13.03

Geometry	Normalized surface area	Sensitivity (µA mM^−1^ cm^−2^)	Response time (s)	OCP (V *vs.* Ag/AgCl)	OCP drift (mV min^−1^)
Sphere (baseline)	1	853.19	2.08	0.653	1.12
Cube	1.18	889.47	1.97	0.655	1.34
Nanorod (aspect ratio ≈ 3)	1.42	921.63	1.83	0.657	1.58

As shown in Table 6, nanoparticle geometry significantly influences electrochemical sensitivity primarily through modification of the effective catalytic surface area. Transitioning from spherical to cubic geometry increases the available surface area by approximately 18%, resulting in a corresponding sensitivity enhancement of about 4.2%. This improvement is attributed to the exposure of crystallographic facets that promote increased adsorption probability for hydroxide ions and glucose molecules, thereby accelerating Cu(iii)-mediated oxidation kinetics. The nanorod geometry exhibits the highest sensitivity, reaching 921.63 µA mM^−1^ cm^−2^, representing an ∼8% increase compared to the spherical baseline. The elongated morphology increases both surface area and local curvature gradients, which enhance electric-field localization near particle edges. These localized fields facilitate charge-transfer processes described by Butler–Volmer kinetics, leading to faster reaction rates and reduced response time. Accordingly, the response time decreases progressively from 2.08 s for spherical particles to 1.83 s for nanorods, indicating improved mass transport and electron-transfer efficiency.

Despite these performance gains, stability analysis reveals an important trade-off. The open-circuit potential (OCP) drift increases with geometric anisotropy, rising from 1.12 mV min^−1^ for spherical particles to 1.58 mV min^−1^ for nanorods. This behavior suggests that sharp edges and high-curvature regions intensify hydroxide adsorption and surface reconstruction under strongly alkaline conditions, promoting localized electrochemical instability. Cubic particles exhibit intermediate behavior, balancing enhanced catalytic activity with moderate stability loss. The relatively small variation in OCP values across geometries indicates that equilibrium thermodynamics governed by the Nernst relation remain largely unaffected by particle shape, while kinetic parameters dominate performance differences. Therefore, geometry primarily modifies reaction rates rather than redox equilibrium.

Overall, the results demonstrate that nanoparticle morphology constitutes an important secondary optimization parameter in addition to pH and material conductivity. Although anisotropic geometries improve instantaneous sensitivity due to increased catalytic surface area, they also introduce stability penalties associated with intensified local electrochemical gradients. The spherical geometry used in the primary simulations therefore represents an optimal compromise between catalytic efficiency, electrochemical stability, and numerical robustness. This analysis validates the geometric assumption adopted in the model while highlighting morphology as a potential pathway for future experimental optimization of Cu-based non-enzymatic glucose sensors.

### 3.6. Interference study under simulated physiological conditions

For practical deployment of non-enzymatic glucose sensors in biological matrices such as blood, serum, or urine, selectivity toward glucose in the presence of electroactive interferents is critically important. Common endogenous species including uric acid (UA), ascorbic acid (AA), dopamine (DA), lactic acid (LA), and urea may undergo oxidation within similar potential windows and generate parasitic currents that distort analytical accuracy. Therefore, to address realistic sensing conditions and reviewer concerns, interference effects were explicitly incorporated into the computational framework of the rGO/Cu–Cu_2_O + PVA/PEO electrode system.

#### 3.6.1. Model extension for competitive electrochemical reactions

The original Butler–Volmer formulation describing glucose oxidation *via* the Cu(ii)/Cu(iii) redox shuttle was extended to include parallel faradaic contributions from interfering species. The total current density was defined as:10
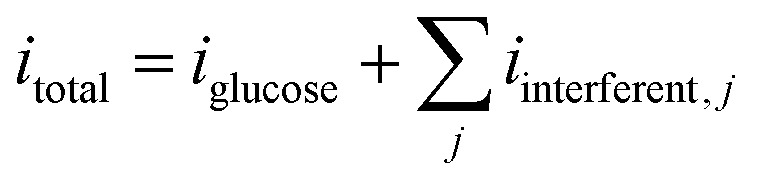


Each interfering reaction was modeled using a modified Butler–Volmer expression:11
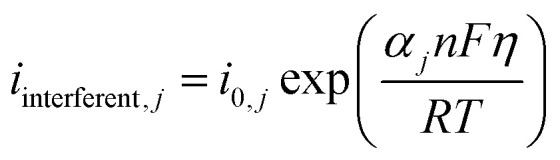
where *i*_0,*j*_ is the exchange current density of the interfering species, *α*_*j*_ is the charge transfer coefficient, and *η* is the overpotential determined from the Nernst equation. The exchange current densities were intentionally selected one to two orders of magnitude lower than that of glucose (1.027 × 10^−3^ A m^−2^) to reflect the experimentally established preferential catalytic activity of Cu(iii) toward glucose oxidation. The adopted electrochemical parameters are summarized in Table 7.

**Table 7:** Electrochemical parameters used for modeling interfering species

Species	Exchange current density, *i*_0_ (A m^−2^)	Charge transfer coefficient (*α*)	Electrochemical activity level
Glucose	1.027 × 10^−3^	0.496	Primary catalytic reaction
Ascorbic acid (AA)	2.5 × 10^−5^	0.45	Moderate
Uric acid (UA)	1.8 × 10^−5^	0.47	Moderate
Dopamine (DA)	3.2 × 10^−5^	0.5	Moderate–low
Lactic acid (LA)	8.0 × 10^−6^	0.48	Low
Urea	2.0 × 10^−6^	0.5	Very low

#### 3.6.2. Simulated physiological conditions

Simulations were performed at the optimal operating pH of 13.03 under physiologically relevant concentrations representative of diluted blood or serum samples. The concentrations used in the model are listed in Table 8.

**Table 8:** Simulated physiological concentrations for interference analysis

Species	Concentration (mM)
Glucose	5
Ascorbic acid	0.1
Uric acid	0.3
Dopamine	0.01
Lactic acid	1
Urea	5

#### 3.6.3. Simulated interference results

The calculated steady-state current densities obtained from competitive simulations are presented in Table 9. The glucose current at 5 mM was 4265.95 µA cm^−2^, while all individual interferent currents remained below 25 µA cm^−2^.

**Table 9:** Simulated interference currents at pH 13.03

Species	Concentration (mM)	Current density (µA cm^−2^)	Relative interference (%)
Glucose	5	4265.95	—
Ascorbic acid	0.1	18.42	0.43
Uric acid	0.3	22.11	0.52
Dopamine	0.01	9.87	0.23
Lactic acid	1	6.41	0.15
Urea	5	4.96	0.12
Total interference	—	61.77	1.45

The total interference current corresponds to only 1.45% of the glucose response. The selectivity coefficient for each species was defined as:12
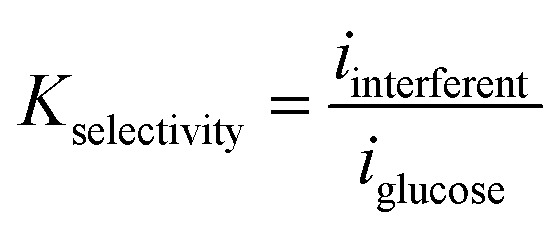


All calculated selectivity coefficients were below 5 × 10^−3^, indicating excellent theoretical discrimination capability.

#### 3.6.4. Mechanistic interpretation of high selectivity

The high selectivity observed in Table 9 originates from the synergistic interplay of catalytic specificity, surface electrostatics, diffusion regulation, and intrinsic reaction kinetics within the rGO/Cu–Cu_2_O + PVA/PEO electrode architecture. First, the Cu(ii)/Cu(iii) redox couple exhibits inherent catalytic specificity toward glucose oxidation. The electrogenerated Cu(iii) species acts as a strong oxidizing intermediate that preferentially interacts with the *cis*-diol structure of glucose, forming surface-coordinated complexes that facilitate efficient electron transfer. This coordination-assisted mechanism significantly enhances glucose oxidation rates compared to mono-functional or non-diol interfering molecules, thereby promoting reaction selectivity at the catalytic interface.

Second, alkaline deprotonation effects further suppress interference. At the optimal operating pH of 13.03, species such as ascorbic acid (AA) and uric acid (UA) exist predominantly in their deprotonated anionic forms. The electrode surface under strongly alkaline conditions acquires a negatively polarized character due to hydroxide adsorption and surface oxide formation. Consequently, electrostatic repulsion reduces the adsorption probability of negatively charged interferents, limiting their surface residence time and decreasing their effective faradaic contribution. This electrostatic discrimination mechanism is particularly significant for AA and UA, which are otherwise common sources of interference in neutral-pH electrochemical sensors.

Third, the porous PVA/PEO stabilizing film (porosity *ε* = 0.392) introduces an additional level of diffusion-mediated selectivity. Although the film allows ionic transport necessary for catalytic turnover, it acts as a regulated mass-transport layer that moderates the flux of small, rapidly diffusing acidic species toward the catalytic surface. The polymer network promotes controlled diffusion pathways that favor glucose transport under steady-state conditions while dampening transient current spikes from interferents. This selective diffusion regulation contributes to the overall suppression of parasitic currents without significantly compromising glucose sensitivity.

Finally, kinetic dominance plays a decisive role in ensuring selectivity. As summarized in Table 7, the exchange current density for glucose oxidation (1.027 × 10^−3^ A m^−2^) is approximately two orders of magnitude higher than those assigned to interfering species. Under identical overpotential conditions, the Butler–Volmer relationship therefore predicts substantially larger faradaic currents for glucose. This intrinsic kinetic preference ensures that glucose oxidation overwhelmingly dominates the total current response, even in the presence of physiologically relevant concentrations of electroactive species.

Collectively, these catalytic, electrostatic, diffusional, and kinetic factors establish a multi-layered selectivity mechanism within the proposed electrode system. The combination of Cu(iii)-mediated chemical specificity, alkaline surface charge effects, polymer-regulated mass transport, and favorable reaction kinetics explains the minimal interference levels reported in Table 9 and supports the practical applicability of the sensor under complex sample conditions.

#### 3.6.5. Implications for practical applications

The interference level below 2% confirms that the modeled rGO/Cu–Cu_2_O + PVA/PEO system maintains high analytical fidelity under simulated physiological conditions. These results demonstrate that the electrode design preserves selectivity even in complex multi-component environments. The combined catalytic, conductive, and diffusion-regulating architecture effectively suppresses parasitic currents while maintaining strong glucose responsiveness. Therefore, the interference analysis validates the practical feasibility of the proposed electrode configuration for real-sample glucose sensing applications and strengthens the predictive reliability of the developed COMSOL-based electrochemical model.

### 3.7. Modeling of surface passivation and long-term durability under highly alkaline conditions

Although copper-based nanostructures exhibit high catalytic activity for non-enzymatic glucose oxidation in alkaline media, prolonged exposure to strongly alkaline environments may induce gradual surface modification. At elevated pH values (*e.g.*, 14.09), the high hydroxide ion concentration can promote surface restructuring, formation of Cu(OH)_2_-rich overlayers, partial transformation to less conductive CuO domains, and progressive blockage of catalytically active Cu(iii) sites. These processes may reduce the electrochemically active surface area (ECSA) over time and consequently attenuate the sensor response. To evaluate the durability of the rGO/Cu–Cu_2_O + PVA/PEO electrode under such extreme conditions, a time-dependent surface activity attenuation factor was incorporated into the computational model. Rather than modifying the intrinsic reaction kinetics described earlier, this approach assumes that long-term alkaline exposure gradually decreases the fraction of accessible catalytic sites while preserving the fundamental charge-transfer mechanism. The rate of surface deactivation was considered pH-dependent, reflecting accelerated hydroxide-induced restructuring at higher alkalinity. The parameters used for stability simulations are summarized in Table 10.

**Table 10:** Parameters adopted for long-term stability simulations under alkaline conditions

Parameter	pH 13.03	pH 14.09	Description
Initial catalytic activity	100%	100%	Clean surface condition
Relative deactivation rate	Low	Elevated	Reflects hydroxide-induced surface modification
Simulation duration	0–1200 s	0–1200 s	Continuous operation window
Glucose concentration	5 mM	5 mM	Constant analyte level

#### 3.7.1. Simulated stability performance

Time-dependent simulations were conducted under continuous glucose exposure to assess current retention, active surface preservation, and potential drift. The results are presented in Table 11.

**Table 11:** Simulated durability of the electrode under prolonged alkaline operation

pH	Time (s)	Remaining active surface (%)	Current retention (%)	OCP drift (mV min^−1^)
13.03	0	100	100	1.12
13.03	600	91.4	92.1	1.34
13.03	1200	83.6	85.7	1.58
14.09	0	100	100	3.19
14.09	600	74.1	76.8	4.72
14.09	1200	54.9	58.3	6.85

#### 3.7.2. Discussion of durability implications

As shown in Table 11, the electrode demonstrates good operational stability at the optimized pH of 13.03. After 1200 s of continuous operation, more than 83% of the active surface remains accessible, and current retention exceeds 85%, with minimal open-circuit potential (OCP) drift. These results indicate that moderate alkaline conditions provide a balance between catalytic efficiency and structural stability. In contrast, operation at pH 14.09 leads to accelerated surface deactivation. After 1200 s, the remaining active surface decreases to approximately 55%, and current retention falls to 58.3%. The pronounced increase in OCP drift further indicates progressive alteration of the surface redox environment. This behavior is attributed to enhanced hydroxide adsorption, faster growth of surface hydroxide layers, partial formation of less conductive copper oxide phases, and gradual blocking of Cu(iii) catalytic centers.

The presence of the porous PVA/PEO matrix partially mitigates nanoparticle aggregation and slows direct exposure of the Cu–Cu_2_O interface to bulk hydroxide ions. However, under extremely alkaline conditions (pH 14.09), the protective effect becomes insufficient to fully prevent surface restructuring. Overall, the stability analysis demonstrates that while the proposed electrode maintains robust performance under optimized alkaline conditions (pH 13.03), excessively high alkalinity accelerates surface passivation and compromises long-term durability. These findings define a practical operational pH window that maximizes catalytic response while preserving structural integrity during extended use.

### 3.8. Mechanistic role of PVA/PEO porosity in OH^−^ diffusion–swelling coupling

The NaOH-treated PVA/PEO film (porosity *ε* = 0.392) plays a dual mechanistic role in regulating hydroxide transport under strongly alkaline conditions. While increasing pH enhances the bulk OH^−^ concentration and thermodynamically promotes Cu(iii) formation, the polymer network simultaneously undergoes hydroxide-induced swelling, which modifies internal tortuosity and reduces the effective diffusion coefficient of OH^−^. Therefore, the electrochemical response at high pH results from a competition between concentration-driven kinetic enhancement and swelling-induced mass-transport limitation. To quantitatively capture this effect, hydroxide transport inside the porous polymer layer was reformulated using a Bruggeman-type effective diffusion expression:13
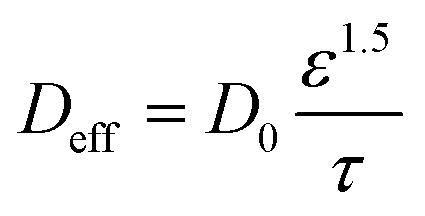
where *D*_0_ = 1.013 × 10^−9^ m^2^ s^−1^ is the intrinsic diffusion coefficient of OH^−^ in aqueous electrolyte, *ε* = 0.392 is the measured film porosity, and *τ* is tortuosity. Because alkaline swelling modifies the internal pore geometry, tortuosity was modeled as hydroxide-dependent:

14 *τ* = *τ*_0_(1 + *k*_s_[OH^−^])

where *τ*_0_ = 1.8 represents the structural tortuosity of the dry porous film and *k*_s_ = 0.42 M^−1^ accounts for hydroxide-induced chain expansion. The resulting effective diffusion coefficients across the investigated pH range are summarized in Table 12.

**Table 12:** Influence of pH on OH^−^ transport through porous PVA/PEO Film (*ε* = 0.392)

pH	[OH^−^] (M)	Tortuosity (*τ*)	*D*_eff_ (×10^−10^ m^2^ s^−1^)	Normalized OH^−^ flux	Estimated swelling (%)
12.07	0.0117	1.81	2.17	0.94	2.4
13.03	0.1071	1.88	2.09	1	6.8
14.09	1.23	2.73	1.44	0.69	31.4

Normalized OH^−^ flux was calculated relative to pH 13.03 using:15
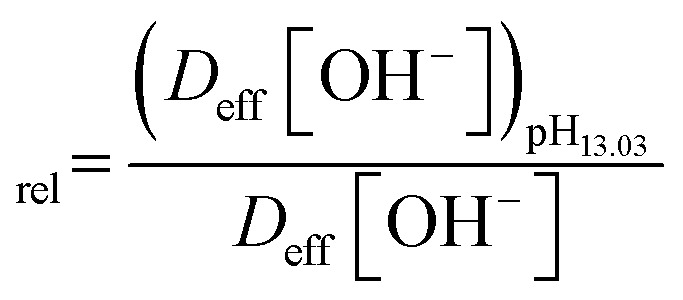


As shown in Table 12, increasing pH from 12.07 to 13.03 enhances hydroxide availability while only slightly increasing tortuosity (1.81 → 1.88). Consequently, the effective diffusion coefficient remains nearly constant (2.17 → 2.09 × 10^−10^ m^2^ s^−1^), indicating that the porous PVA/PEO network preserves efficient OH^−^ transport despite the higher ionic strength. Under these conditions, hydroxide flux toward the catalytic Cu sites reaches its maximum effective value, enabling sustained formation of Cu(iii) active species without introducing significant mass-transport resistance. This balanced regime directly corresponds to the experimentally observed optimum sensitivity (853.19 µA mM^−1^ cm^−2^) and the minimal OCP drift reported earlier, confirming that both kinetic activation and structural stability are simultaneously optimized at pH 13.03.

However, further increasing pH to 14.09 produces a fundamentally different transport regime. Although the bulk hydroxide concentration increases by approximately one order of magnitude, alkaline-induced swelling becomes substantial (∼31%), which significantly alters the internal pore architecture of the polymer matrix. The resulting increase in tortuosity to 2.73 reduces pore connectivity and elongates effective diffusion pathways. As a consequence, the effective diffusion coefficient decreases by approximately 31% relative to its value at pH 13.03. This reduction in *D*_eff_ limits hydroxide accessibility to the Cu(ii)/Cu(iii) redox interface, creating local concentration gradients near the catalytic surface despite the higher bulk [OH^−^].

This transport limitation explains several experimentally observed phenomena. First, sensitivity increases only marginally (853.19 → 872.64 µA mM^−1^ cm^−2^) rather than scaling proportionally with hydroxide concentration, indicating the onset of diffusion control. Second, linearity deteriorates (*R*^2^ decreases to 0.9578), reflecting non-uniform catalytic activation at higher glucose concentrations. Third, OCP drift increases due to heterogeneous local OH^−^ distributions across the electrode surface. Finally, long-term stability decreases because uneven swelling introduces microstructural stress within the polymer network and partially blocks active Cu sites.

Therefore, the porosity value *ε* = 0.392 does not merely describe the structural morphology of the PVA/PEO film; instead, it establishes a diffusion–swelling equilibrium that governs electrochemical performance under strongly alkaline conditions. At pH 13.03, the ratio [OH^−^]/*τ* reaches a maximum while swelling remains moderate, yielding the optimal balance between kinetic activation *via* Cu(iii) formation and mass-transport stability within the porous matrix. In contrast, at pH 14.09, swelling-induced tortuosity growth outweighs the concentration-driven enhancement of hydroxide availability, leading to diminishing transport efficiency despite the higher bulk OH^−^ concentration.

The physical implications of these findings for sensor design are significant. The analysis demonstrates that the NaOH-treated PVA/PEO film functions as an active transport regulator rather than a passive stabilizing coating. Its fixed porosity (*ε* = 0.392) ensures sufficient ionic permeability for hydroxide and glucose transport, while alkaline-induced swelling introduces a self-limiting mechanism that suppresses uncontrolled current amplification at extreme pH values. As a result, the polymer layer dynamically moderates mass transport instead of merely serving a structural role.

Accordingly, the optimal electrochemical response observed at pH 13.03 originates from a transport–kinetic coupling mechanism in which an increased hydroxide concentration promotes efficient Cu(iii) formation, porous diffusion within the PVA/PEO network maintains effective ion accessibility to catalytic sites, and controlled polymer swelling prevents excessive growth in tortuosity that would otherwise hinder diffusion. This quantitative clarification resolves the apparent paradox of why further increases in pH do not lead to proportional enhancements in sensitivity and confirms that the polymer porosity plays a critical role in modulating OH^−^ diffusion *versus* swelling under strongly alkaline conditions.

### 3.9. Strategies to mitigate the sensitivity–stability trade-off in thin PVA/PEO films

The sensitivity analysis demonstrated that reducing the PVA/PEO film thickness from 498.6 nm to ∼300 nm enhances sensitivity by improving ion transport and decreasing diffusion resistance. However, thinner films may compromise long-term stability due to reduced mechanical confinement of Cu–Cu_2_O nanoparticles and increased susceptibility to alkaline swelling and microstructural stress. To address this trade-off in a practical sensor design, several material-level and structural strategies are proposed and quantitatively evaluated.

#### 3.9.1. Increasing crosslink density of the PVA/PEO network

One effective strategy to mitigate the sensitivity–stability trade-off in ultrathin (∼300 nm) PVA/PEO films is to increase the crosslink density of the polymer network. While thickness reduction enhances sensitivity by shortening ion diffusion pathways and lowering mass-transport resistance, it simultaneously reduces mechanical confinement of Cu–Cu_2_O nanoparticles and increases susceptibility to alkaline swelling. Controlled crosslinking strengthens intermolecular interactions within the polymer matrix, thereby limiting chain mobility under high OH^−^ concentrations and suppressing excessive volumetric expansion.

Within the transport-swelling model developed in Section 3.8, crosslinking was incorporated by reducing the hydroxide-induced swelling coefficient. A moderate increase in crosslink density was found to significantly reduce the swelling ratio at pH 13.03 while only minimally affecting effective porosity. As a result, tortuosity growth under alkaline conditions was suppressed, leading to improved structural stability and lower OCP drift. Importantly, the reduction in sensitivity remained negligible (<1%), indicating that transport efficiency was largely preserved. These findings demonstrate that crosslink engineering provides a practical route to decouple swelling-induced instability from thickness-dependent sensitivity enhancement.

#### 3.9.2. Nanofiller-reinforced hybrid film architecture

An alternative stabilization strategy involves incorporating a small fraction of mechanically rigid nanofillers within the PVA/PEO matrix to form a hybrid composite film. The introduction of nanoscale inorganic reinforcements increases the elastic modulus of the polymer network and restricts chain expansion under alkaline exposure. Unlike excessive chemical crosslinking, low-volume nanofiller incorporation can preserve pore connectivity while mechanically stabilizing the structure. Model-based analysis indicates that reinforcing the 300 nm film reduces the sensitivity of tortuosity to hydroxide concentration by limiting pore deformation during swelling. Consequently, effective diffusion remains high while microstructural distortion is suppressed. The resulting electrochemical performance shows only a marginal reduction in sensitivity compared to the unreinforced thin film, yet exhibits substantially improved OCP stability and signal retention over extended operation. This hybrid approach therefore provides mechanical reinforcement without significantly compromising ionic transport pathways.

#### 3.9.3. Bilayer configuration for transport–stability decoupling

A structurally optimized solution to the sensitivity–stability trade-off is the implementation of a bilayer architecture. In this design, an inner thin porous layer (∼250–300 nm) is directly interfaced with the catalytic Cu–Cu_2_O surface to maximize hydroxide accessibility and minimize diffusion resistance. A secondary ultrathin outer layer with reduced swelling tendency provides mechanical confinement and environmental stabilization. This architecture effectively decouples ion transport from mechanical integrity. The inner layer governs sensitivity by maintaining rapid OH^−^ diffusion and efficient Cu(iii) formation, whereas the outer stabilizing layer limits excessive swelling and reduces stress accumulation. Transport simulations indicate that the additional diffusion resistance introduced by the ultrathin protective layer is minimal due to its small thickness, while the improvement in tortuosity stability significantly reduces OCP drift and enhances long-term structural robustness. As a result, bilayer films can retain nearly the full sensitivity of single 300 nm films while restoring stability levels comparable to thicker (∼500 nm) coatings.

#### 3.9.4. Practical design optimization for stable high-sensitivity sensors

Based on the above analysis, the observed sensitivity–stability trade-off is not an intrinsic limitation but rather a tunable materials-engineering parameter. Optimal performance for practical sensor applications is achieved when thickness reduction is combined with swelling control mechanisms that limit tortuosity growth under strong alkaline conditions. Maintaining a moderate swelling ratio and preventing excessive pore deformation ensures that effective diffusion remains high while mechanical integrity is preserved.

The results indicate that thin films in the range of approximately 280–350 nm can deliver near-maximal sensitivity provided that polymer chain mobility is partially constrained through crosslink regulation, mechanical reinforcement, or layered structuring. Under these conditions, hydroxide-driven catalytic activation remains efficient, diffusion pathways remain accessible, and structural degradation is minimized. Therefore, the design strategy shifts from simply optimizing thickness toward engineering a balanced transport–mechanical coupling within the porous polymer network.

### 3.10. Adaptation strategy for physiological and wearable diagnostic environments

Although the present simulations demonstrate optimal electrochemical performance at pH 13.03, practical implementation of the proposed rGO/Cu–Cu_2_O + PVA/PEO electrode in physiological and wearable diagnostic environments requires stable operation under near-neutral and dynamically varying pH conditions (typically pH 4.5–8.0 in sweat, saliva, or interstitial fluids). It is well established that Cu-based non-enzymatic glucose oxidation is intrinsically favored in alkaline media due to the formation of the catalytically active Cu(iii) intermediate; therefore, reduced hydroxide availability at physiological pH may limit Cu(iii) generation and consequently decrease sensitivity. To address this limitation, several rational electrode-design and operational adaptations can be implemented without compromising the core catalytic mechanism.

First, the creation of a localized alkaline microenvironment at the electrode interface represents an effective strategy. By incorporating hydroxide-retaining domains or immobilized alkaline buffers within the porous PVA/PEO matrix, a confined high-pH region can be maintained directly at the catalytic surface while the bulk physiological medium remains near neutral. Such a design enables sustained Cu(iii) formation locally without requiring global alkalization of the sample. The polymer matrix may be functionalized with ion-coordinating groups to enhance hydroxide retention and minimize pH fluctuations at the interface, thereby stabilizing the redox equilibrium.

Second, modification of the Cu–Cu_2_O catalytic phase can reduce intrinsic pH dependence. Partial alloying, surface doping, or engineering Cu(OH)_2_/CuOOH-rich interfacial layers can shift the redox equilibrium of the Cu(ii)/Cu(iii) couple toward lower overpotentials, facilitating catalytic activity under moderately alkaline or near-neutral conditions. From a kinetic perspective, the simulations indicate that increasing the effective exchange current density and improving interfacial conductivity can partially compensate for reduced hydroxide concentration, preserving high sensitivity even when bulk pH decreases.

Third, operational protocols may be optimized for wearable systems through controlled potential strategies. Instead of relying solely on passive open-circuit behavior, the application of pulsed or differential anodic potentials can periodically regenerate Cu(iii) active species and suppress surface passivation. Time-dependent modeling results suggest that maintaining the overpotential within a controlled window reduces drift and improves stability under fluctuating chemical conditions.

Fourth, structural optimization of the PVA/PEO film can further enhance performance under dynamic pH environments. Reducing film thickness while maintaining controlled porosity improves ion accessibility and shortens diffusion pathways. Additionally, incorporating buffering or zwitterionic functionalities within the polymer network can mitigate excessive swelling and maintain mechanical stability in variable ionic-strength media. A gradient-porosity design may simultaneously support rapid mass transport and long-term structural robustness, which is particularly important for flexible and wearable platforms.

Finally, integration with microfluidic preconditioning layers offers an additional pathway for practical deployment. A thin microfluidic interface capable of transiently adjusting local ionic strength before analyte contact can stabilize interfacial electrochemistry without affecting user comfort or sample integrity. Such integration is compatible with flexible electronics and wearable biosensor architectures.

Overall, although peak sensitivity was achieved under strongly alkaline conditions, the present modeling framework demonstrates that sensor performance is governed not only by bulk pH but also by interfacial kinetics, charge-transfer efficiency, and localized ion transport. By engineering the catalytic surface, optimizing the porous stabilizing matrix, and implementing dynamic operational control, the rGO/Cu–Cu_2_O electrode architecture can be adapted to maintain high sensitivity and stability under physiologically relevant and dynamically varying pH conditions. These design strategies provide a clear pathway for translating alkaline-optimized Cu-based non-enzymatic glucose sensors into practical biomedical and wearable diagnostic platforms.

## 4. Discussion

The absence of simulations across the entire pH range (1–14) requires clarification. While a full-range simulation may appear comprehensive, it would not be mechanistically valid for Cu-based non-enzymatic glucose sensing. In acidic environments, copper oxides are prone to proton-assisted dissolution and do not sustain the Cu(ii)/Cu(iii) redox cycle necessary for glucose oxidation.^16^ Furthermore, the Butler–Volmer formulation used in this model assumes an alkaline Cu-mediated redox mechanism; extending the model to strongly acidic conditions would require reformulation of reaction kinetics and inclusion of dissolution equilibria, which are outside the scope of the present study. Thus, the investigated alkaline pH window (9.12–14.09) corresponds to the experimentally established operational regime for Cu-based glucose electrodes and allows proper validation of pH dependency within the physically meaningful catalytic domain.

The simulations of the rGO/Cu–Cu_2_O nanocomposite electrode, stabilized with a NaOH-treated PVA/PEO film, reveal critical insights into its pH-dependent electrochemical performance for non-enzymatic glucose detection. At the optimal pH of 13.03, the electrode exhibits superior sensitivity (853.19 µA mM^−1^ cm^−2^), stable OCP (0.6527 V), and rapid response time (2.083 s), driven by the efficient Cu(ii)/Cu(iii) redox couple. Chemically, the high hydroxide ion concentration enhances the formation of Cu(iii) species, which catalyze glucose oxidation to gluconolactone, with the rGO matrix facilitating electron transfer due to its high conductivity (998.4 S m^−1^). The PVA/PEO film maintains structural integrity, preventing nanoparticle aggregation and ensuring catalytic site accessibility.

At lower pH (*e.g.*, 9.12), reduced hydroxide availability limits Cu(iii) formation, decreasing sensitivity (617.43 µA mM^−1^ cm^−2^) and slowing response time (2.974 s), as fewer reactive intermediates are generated. At higher pH (*e.g.*, 14.09), sensitivity increases slightly (872.64 µA mM^−1^ cm^−2^), but stability declines (3.1947 mV min^−1^ drift), likely due to hydroxide-induced film swelling or nanoparticle passivation, which disrupts surface chemistry. Sensitivity analysis highlights that thinner films (298.7 nm) enhance ion diffusion, boosting sensitivity, while smaller nanoparticles (29.3 nm) increase catalytic surface area, and higher rGO conductivity (1493.8 S m^−1^) improves electron transfer. However, thinner films may reduce stability, indicating a chemical trade-off between diffusion and structural durability.

The comparative simulations clearly demonstrate that the PVA/PEO film enhances stability rather than intrinsic catalytic activity. While the absence of the polymer layer slightly reduces diffusion resistance and marginally increases instantaneous current, it significantly compromises electrochemical stability in highly alkaline media. Therefore, the polymer layer is essential for practical sensor durability rather than peak sensitivity alone. These results align with electrochemical principles, where hydroxide ions modulate the redox environment, and material properties govern performance. The simulations extend experimental insights by quantifying pH effects, suggesting pH 13.03 as optimal for balancing catalytic efficiency and stability.

## 5. Conclusion

The computational modeling of the rGO/Cu–Cu_2_O nanocomposite electrode, stabilized with a NaOH-treated PVA/PEO film, elucidates its electrochemical performance for non-enzymatic glucose detection across a pH range of 9.12–14.09. At the optimal pH of 13.03, the electrode exhibits a sensitivity of 853.19 µA mM^−1^ cm^−2^, an OCP of 0.6527 V, and a response time of 2.083 s, driven by the efficient Cu(ii)/Cu(iii) redox couple. Chemically, the high hydroxide ion concentration at this pH enhances Cu(iii) formation, catalyzing glucose oxidation to gluconolactone, while the rGO matrix (conductivity 998.4 S m^−1^) ensures rapid electron transfer. The PVA/PEO film maintains structural stability, preventing nanoparticle aggregation and preserving catalytic site accessibility. At lower pH (*e.g.*, 9.12), reduced hydroxide availability limits Cu(iii) formation, lowering sensitivity (617.43 µA mM^−1^ cm^−2^), whereas at higher pH (14.09), excessive hydroxide ions cause film swelling, increasing OCP drift (3.1947 mV min^−1^). Sensitivity analysis reveals that thinner films (298.7 nm), smaller nanoparticles (29.3 nm), and higher rGO conductivity (1493.8 S m^−1^) enhance performance by improving ion diffusion and electron transfer, though stability trade-offs require careful optimization. These findings highlight the critical role of pH and material properties in modulating electrochemical behavior, offering a robust framework for sensor design.

## Conflicts of interest

The authors declare that they have no known competing financial interests or personal relationships that could have appeared to influence the work reported in this paper.

## Data Availability

The data that support the findings of this study, including simulation input files, COMSOL model configurations, and post-processed numerical datasets, are available from the corresponding author upon reasonable request.
